# Pathophysiological evaluation of the *LRRK2* G2385R risk variant for Parkinson’s disease

**DOI:** 10.1038/s41531-022-00367-y

**Published:** 2022-08-05

**Authors:** Toshiki Tezuka, Daisuke Taniguchi, Mariko Sano, Tomoyo Shimada, Yutaka Oji, Taiji Tsunemi, Aya Ikeda, Yuanzhe Li, Hiroyo Yoshino, Jun Ogata, Kahori Shiba-Fukushima, Manabu Funayama, Kenya Nishioka, Yuzuru Imai, Nobutaka Hattori

**Affiliations:** 1 Department of Neurology, Juntendo University School of Medicine, Tokyo, 113-8421 Japan; 2 Department of Neurology, Keio University School of Medicine, Tokyo, 160-8582 Japan; 3 Research Institute for Diseases of Old Age, Juntendo University Graduate School of Medicine, Tokyo, 113-8421 Japan; 4 Department of Research for Parkinson’s Disease, Juntendo University Graduate School of Medicine, Tokyo, 113-8421 Japan; 5 Department of Drug Development for Parkinson’s Disease, Juntendo University Graduate School of Medicine, Tokyo, 113-8421 Japan; 6 Center for Genomic and Regenerative Medicine, Graduate School of Medicine, Juntendo University, Tokyo, 113-8421 Japan; 7 Neurodegenerative Disorders Collaborative Laboratory, RIKEN Center for Brain Science, 2-1-Hirosawa, Wako-shi, Saitama, 351-0198 Japan

**Keywords:** Parkinson's disease, Proteins, Risk factors

## Abstract

Missense variants in *leucine-rich repeat kinase 2 (LRRK2)* lead to familial and sporadic Parkinson’s disease (PD). The pathological features of PD patients with LRRK2 variants differ. Here, we report an autopsy case harboring the LRRK2 G2385R, a risk variant for PD occurring mainly in Asian populations. The patient exhibited levodopa-responsive parkinsonism at the early stage and visual hallucinations at the advanced stage. The pathological study revealed diffuse Lewy bodies with neurofibrillary tangles, amyloid plaques, and mild signs of neuroinflammation. Biochemically, detergent-insoluble phospho-α-synuclein was accumulated in the frontal, temporal, entorhinal cortexes, and putamen, consistent with the pathological observations. Elevated phosphorylation of Rab10, a substrate of LRRK2, was also prominent in various brain regions. In conclusion, G2385R appears to increase LRRK2 kinase activity in the human brain, inducing a deleterious brain environment that causes Lewy body pathology.

## Introduction

*Leucine-rich repeat kinase 2* (*LRRK2*) has been identified as the causative gene for *PARK8-*linked Parkinson’s disease (PD)^1,2^. The gene product of LRRK2 contains multiple protein domains, including armadillo repeat, ankyrin repeat, leucine-rich repeat (LRR), Ras-of-complex (ROC), C-terminal of Roc (COR), kinase, and WD40 domains^2,3^. Disease-linked missense variants have been found in some of these domains^4^. LRRK2 variants are also common risk factors for sporadic PD^5^. Among them, the Gly2385Arg (G2385R) variant, which is located in the WD40 domain, is the most common PD risk factor in the Asian populations, with a 2-fold risk of PD in the Chinese population^6,7^. In ethnic Chinese and Japanese populations, the G2385R variant has a frequency of 6.7–11.6% in sporadic PD patients and 3.6–5.6% in healthy individuals^6,8^. Compared with patients experiencing PD who do not carry the LRRK2 G2385R, the carriers have a higher frequency of family history^9^; longer disease duration^10^; lower age of onset^7^; and higher proportion of postural instability and gait disorder phenotype^11^. Additionally, carriers are characterized by levodopa-induced complications, including motor fluctuations and dyskinesia^11,12^; specific non-motor phenotypes including rapid eye movement sleep behavior disorders (RBDs) and fatigue^11,13^; and a higher Mini-Mental State Examination (MMSE) score^11^. However, a recent meta-analysis reported no significant differences in the sex distribution, age at onset, initial symptoms, motor symptoms, depression, levodopa-equivalent dose, and related complications between LRRK2 G2385R-carrier and LRRK2 G2385R-noncarrier PD patients. This suggests that most of the clinical characteristics of PD patients with LRRK2 G2385R variant are similar to those of PD patients without LRRK2 G2385R variant among Asian PD patients^14^.

Although various functions have been proposed for LRRK2, recent structural biological analyses suggest that LRRK2 is involved in microtubule dynamics and microtubule-dependent transport^15,16^. The WD40 domain is responsible for LRRK2-induced neurotoxicity^17,18^ and has been shown to mediate LRRK2 interaction with microtubules and synaptic vesicles^19,20^, enhancing the microtubule association of LRRK2 and forming a skein-like structure in cells^19^. An in vitro study indicated that PD-associated disease variants in the WD40 domain, including G2385R variant, primarily compromise dimer formation^21^. Meanwhile, G2385R moderately enhances LRRK2 kinase activity^21^ and reduces the skein-like formation by LRRK2 specific kinase inhibitors^15,22^. However, various other studies reported that G2385R variant did not alter or reduce LRRK2 kinase activity in vitro or in cultured cells^22,23^. A part of Rab family proteins, which includes Rab8 and Rab10, have been reported as physiological substrates of LRRK2 kinase, but the effects of their phosphorylation on neurons and glia await further analysis^24,25^.

Heterogeneous brain pathology is a feature of *LRRK2*-linked PD^2,26,27^; as such, knowing whether there is a correlation between LRRK2 variants in each domain and specific pathology is an important approach that will contribute to our understanding of the pathogenesis of *LRRK2*-linked PD. Although a skin biopsy study reports that synucleinopathy is associated with LRRK2 G2385R^28^, no brain autopsy of PD with LRRK2 G2385R has been reported. Here, we report a PD case with LRRK2 G2385R wherein a typical diffuse Lewy body disease pathology with mild gliosis, possibly caused by increased LRRK2 kinase activity in the brain was observed.

## Results

### Case presentation

**Fig. 1:**
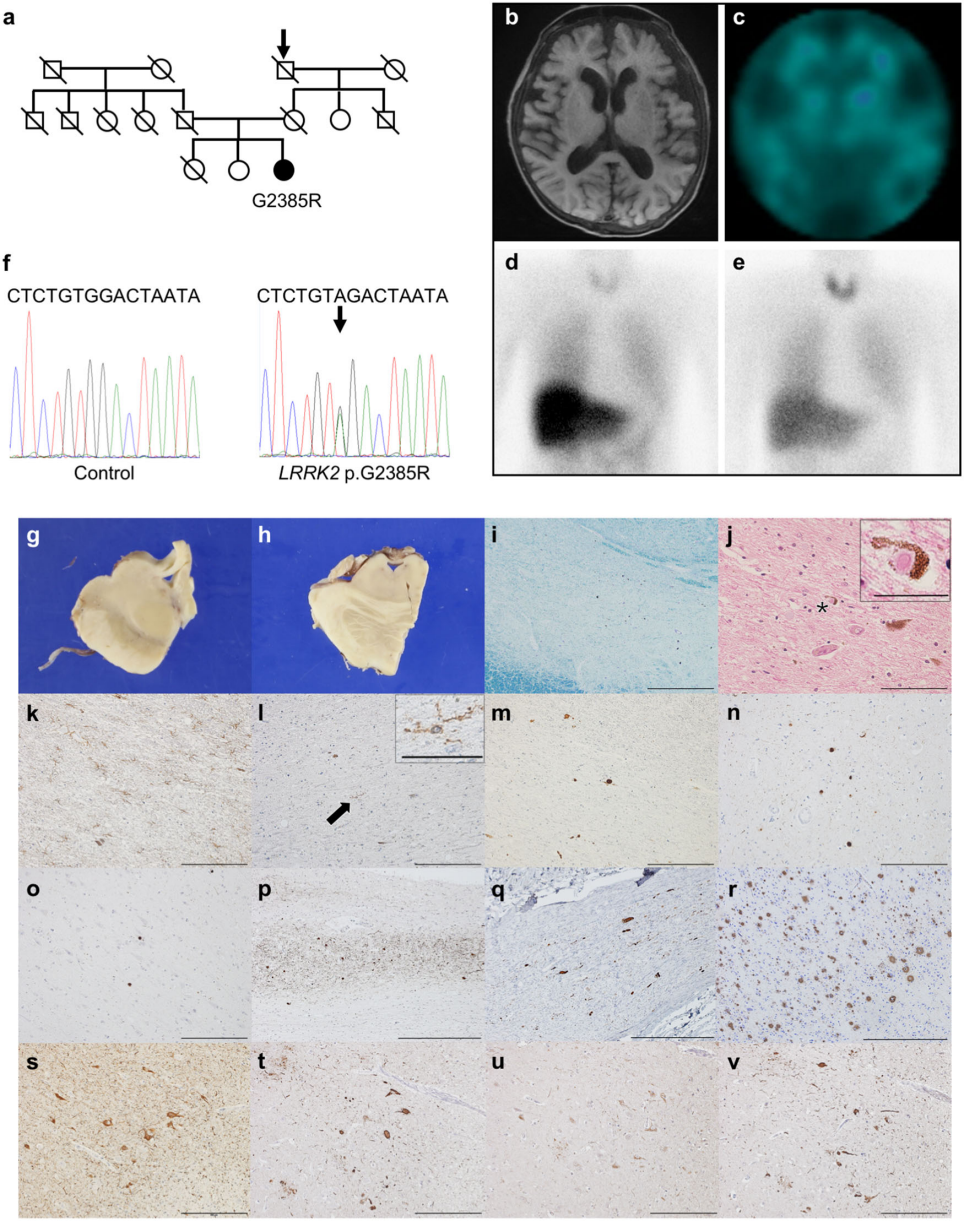
Diffuse Lewy body pathology in a LRRK2 G2385R case. **a** Family tree harboring LRRK2 G2385R. Black, PD; diagonal lines, deceased; Arrow, family member experiencing tremors when writing, but undiagnosed with PD. **b** No brain MRI abnormalities. **c** DAT-SPECT shows decrease DAT densities. [^123^I] MIBG myocardial scintigraphy reveals H/M ratio decreasing from 1.46 at early (**d**) to 1.35 at delay (**e**). **f** Sanger sequencing detects c.7153 G > A (p.G2385R) mutation in LRRK2 exon 48. Macroscopic picture of the midbrain (**g**) and locus coeruleus (**h**). Marked depigmentation in the substantia nigra (SN) (**i**). The brainstem-type Lewy body (asterisk) in the SN; high-magnification image is shown in the inset (**j**). Mild gliosis (**k**) and ramified microglia (arrow) in the SN; high-magnification image in the inset (**l**). The severity of Lewy pathology is moderate in the SN (**m**), severe in the amygdala (**n**), and moderate in the middle frontal cortex (**o**). Lewy pathology is also detected in the olfactory bulb (**p**) and thoracic sympathetic ganglia (**q**). Abundant cored and diffuse senile plaques in the middle temporal cortex (**r**). Neurofibrillary tangles and threads in the parahippocampal gyrus, stained using AT8 (**s**), anti-3-repeat tau (**t**), anti-4-repeat tau (**u**) antibodies. Strongly stained Tau inclusion by AD-specific tau antibody (**v**). Klüver-Barrera (**i**), hematoxylin and eosin (**j**), anti-glial fibrillary acidic protein (**k**), anti-Iba1 (**l**), anti-phospho-αS (**m**–**q**), methenamine-silver staining (**r**), AT8 (**s**), RD3 (**t**), RD4 (**u**), anti-4-repeat tau (**v**). Scale bars: 1 mm (**i**), 500 µm (**p**–**r**), 200 µm (**k**–**o**, **s**–**v**), 100 µm (**j**), 25 µm (**j**, **l**, inset).

The patient was the oldest of three sisters. There was no consanguinity in her family. Though her maternal grandfather had writing tremor, no other members in the family developed PD. This patient, indicated as black symbols in the family tree, was clinically diagnosed with typical PD (Fig. 1a). The patient had a heterozygous mutation of G2385R in *LRRK2*. At an age of 42 years, she noticed right-sided dominant tremor in her upper limb and was diagnosed with PD. Following diagnosis, her motor symptoms showed a good response to levodopa/carbidopa, trihexyphenidyl, amantadine, and ropinirole for about 25 years. Her parkinsonism slowly progressed and wearing off phenomenon emerged by 67 years of age. At 68 years, she started to have prominent visual hallucinations and dyskinesia, leading to frequent falls. At 70 years, she underwent genetic testing and cognitive examination, revealing mildly impaired cognitive function, with an MMSE score of 25/30. Her non-motor symptoms included delusions, RBD, sudden sleepiness, restless legs syndrome, constipation, orthostatic hypotension, urinary disturbance, and paresthesia in her lower extremities. After turning 68 years old, she lost 8 kg body weight in two years. Her motor symptom improved by sleeping (sleep benefit). Additionally, she did not suffer from olfactory dysfunction, depression, gaze palsy, dystonia, and cerebellar ataxia. Brain magnetic resonance imaging (MRI) indicated no abnormalities (Fig. 1b). Dopamine transporter single-photon emission computed tomography (DAT-SPECT) showed a severe decrease in DAT densities (Fig. 1c). [^123^I] metaiodobenzylguanidine (MIBG) myocardial scintigraphy revealed a decreasing rate of heart to mediastinum (H/M) ratio, with 1.46 and 1.35 as early and delay values, respectively (normal value, over 2.2) (Fig. 1d, e). At 72 years, she developed difficulty in walking and was repeatedly admitted to our hospital due to a urinary tract infection. Three months before her death, when she left our hospital after the treatment of the urinary tract infection, her dose of levodopa/carbidopa was increased from 150 mg four times to 200 mg six times a day, and she was able to walk with a little aid (Hoehn Yahr Stage IV, MDS-UPDRS part3: 48 points). However, due to her prolonged hospitalization, her cognitive function worsened and she became bedridden after the discharge. Subsequently, her urinary tract infection recurred, and she could barely take her medications. When she was admitted to our hospital, she was in a coma and suffered from repeated hypoglycemia caused by sepsis (Hoehn Yahr Stage V, MDS-UPDRS part3: 96 points). Although she was provided intensive care and antibiotics, disseminated intravascular coagulation gradually progressed and she died of urosepsis within one week.

### Genetic analysis

Sanger sequencing revealed a heterozygous mutation of LRRK2 G2385R in this patient (Fig. 1f). We also confirmed a heterozygous mutation of LRRK2 R1441H^29^ or I2020T^30^ in two patients for comparison in this study (Supplementary Table 1). There were no pathogenic variants and risk variants related to familial PD except LRRK2 variants in the G2385R patient and the other two cases (see Clinical and genetic analysis in Methods).

### Neuropathological findings

The brain was slightly lighter (1190 g) than the average brain without apparent atrophy of the cerebrum, brainstem, and cerebellum. The substantia nigra (SN) and the locus coeruleus (LC) were markedly depigmented (Fig. 1g, h). Marked neuronal loss (Fig. 1i), mild gliosis with the brainstem-type Lewy bodies (LBs) (Fig. 1j, k), and sparse microglia with a ramified appearance (Fig. 1l) were observed in the SN pars compacta. The LC and the dorsal nucleus of vagal nerve showed moderate neuronal loss. Cortical-type LBs were detected in the limbic system and cerebral cortex. Immunohistochemistry for α-synuclein (αS) phosphorylated at Ser129 revealed LBs and Lewy neurites in the brainstem (Fig. 1m), limbic system (Fig. 1n), and cerebral cortex (Fig. 1o), as well as in the olfactory nerve (Fig. 1p) and thoracic sympathetic ganglia (Fig. 1q). Consistent with the result of MIBG myocardial scintigraphy, the reduction of anti-tyrosine hydroxylase (TH)-positive signals and accumulation of phospho-Ser129 αS-positive inclusions were observed in the sympathetic fibers of the anterior wall of the left ventricle (Supplementary Fig. 1)^31^. The severity of Lewy pathology corresponds to diffuse neocortical type. Senile plaques were frequently present in the cerebral cortex but sparsely in the limbic system and striatum, consistent with Braak senile plaque stage C and Thal phase 3 (Fig. 1r). Neurofibrillary tangles (NFTs) and threads were detected from the hippocampus to the temporal gyrus, parahippocampal gyrus, and amygdala as well as mildly in the insular cortex, corresponding to Braak NFT stage IV and AT8 stage IV (Fig. 1s). NFTs and threads had strong and weak immunoreactivity to RD3 (Fig. 1t) and RD4 (Fig. 1u), respectively. Meanwhile, these tau inclusions were strongly positive with anti-4-repeat tau antibody, which specifically recognized Alzheimer’s disease (AD)-type 4-repeat tau isoform (Fig. 1v)^32^. These changes corresponded to an “intermediate” stage of AD pathologic change (A2B2C3) (Supplementary Table 1)^33^. There were no phospho-TAR DNA-binding protein 43-positive inclusions or other pathology such as cerebrovascular disorders. Overall, the present case was neuropathologically diagnosed as diffuse Lewy body disease with AD pathology.

### Biochemical characterization of LRRK2 G2385R brain autopsy

**Fig. 2:**
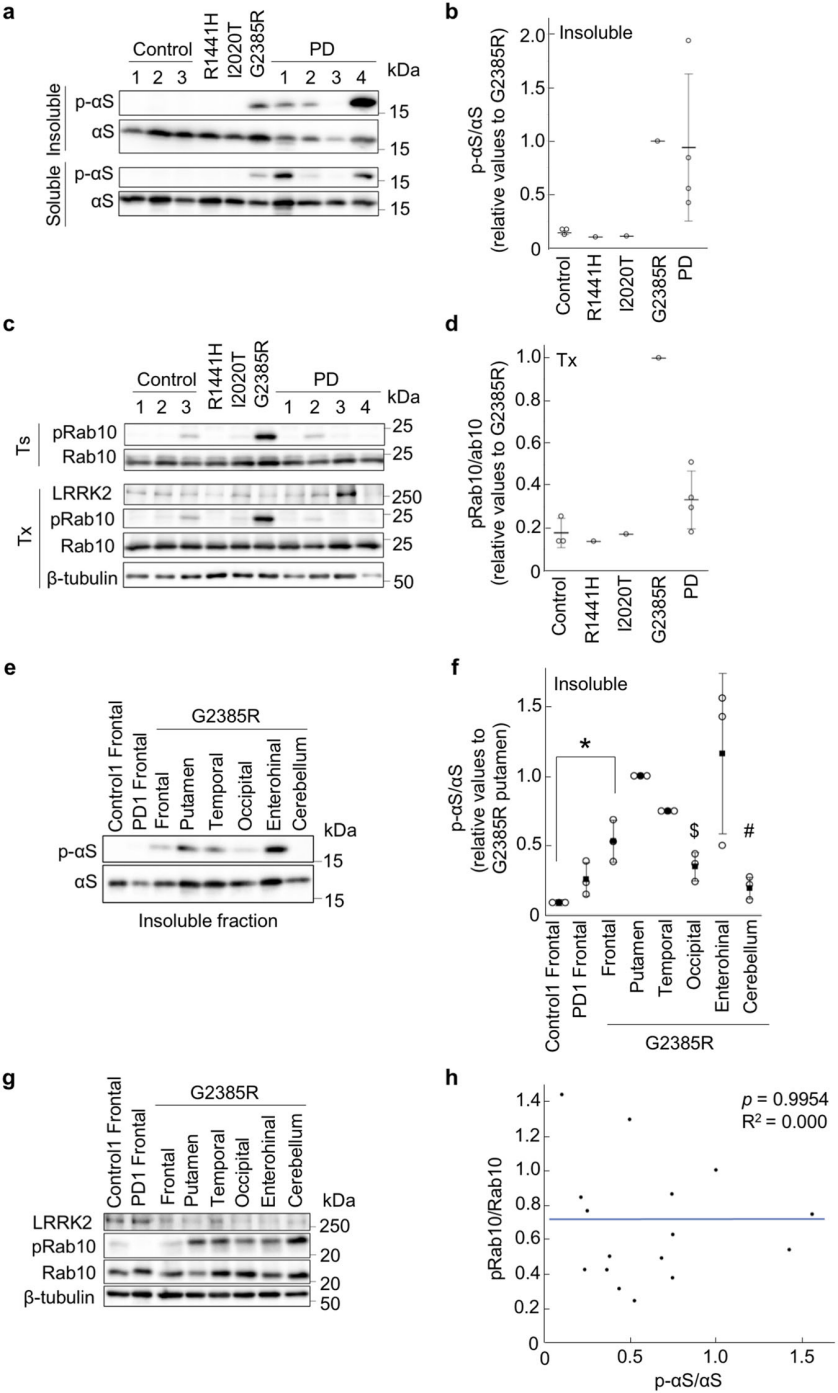
Increased levels of phospho-Rab10 by LRRK2 G2385R is not associated with phospho-αS accumulation. **a** αS and phospho-αS (p-αS) in sarkosyl-soluble and insoluble fractions of brain autopsies. **b** Graph representing quantification of sarkosyl-insoluble p-αS normalized with sarkosyl-insoluble αS as shown in **a**. Dots represent average from three technical replications. Horizontal lines and error bars indicate average and standard deviation, respectively. **c** Phospho-Rab10 (pRab10) in Tris buffer (Ts) and Triton X-100 (Tx)-soluble fractions. ß-tubulin serves as a loading control. **d** Graph representing quantification of pRab10 in Triton X-100 (Tx)-soluble fraction as shown in **c**. Dots represent average from three technical replications. Horizontal lines and error bars indicate average and standard deviation, respectively. **e** Sarkosyl-insoluble αS and p-αS levels in different brain regions of the LRRK2 G2385R case. Extracts of the frontal lobes in control-1 and PD-1 served as controls. **f** Graph represents quantification of sarkosyl-insoluble p-αS normalized with sarkosyl-insoluble αS as shown in **e**. *p < 0.0001 *vs*. control-1 frontal by Dunnett’s test. ^*#*^*p* = 0.0075; ^$^*p* = 0.0293 *vs*. G2385R putamen by Dunnett’s test. *n* = 3 technical replications. **g** Tx-soluble LRRK2, Rab10, and pRab10 levels in different brain regions of the LRRK2 G2385R case. **h** Correlation analysis between the levels of sarkosyl-insoluble p-αS normalized with αS and those of Tx-soluble pRab10 normalized with Rab10 in the LRRK2 G2385R case. *P-*value and coefficient of determination are calculated using Pearson’s test with the data in **e** and **g**. The blue line represents linear regression line.

The clinical information of the brain autopsies used in the biochemical study are summarized in Supplementary Table 1. The levels of phospho-Ser129 αS of the putamen in sarkosyl-insoluble fraction of LRRK2 G2385R were higher than those in the other LRRK2 variant or non-PD control group, whereas they were comparable to those in the sporadic PD control group (Fig. 2a, b). The phospho-T73 Rab10 levels in the putamen appeared to be increased in LRRK2 G2385R brain, based on the phospho-Rab10 levels of other groups (Fig. 2c, d). LRRK2 expression was also confirmed in all cases (Fig. 2c). We further examined the levels of phospho-αS and phospho-Rab10 in different brain regions of the patient with LRRK2 G2385R, including the frontal, temporal, occipital, entorhinal cortexes, and cerebellum, comparing with those in the frontal cortex of non-PD control-1 and PD-1 groups (Fig. 2e–g). Phospho-αS was accumulated in the frontal, temporal, entorhinal cortexes, and the putamen of LRRK2 G2385R, consistent with pathological observations (Fig. 2f). Marked phosphorylation of Rab10 was detected in all analyzed regions except for the frontal cortex in the LRRK2 G2385R brain (Fig. 2g). However, there was no correlation between phospho-αS and phospho-Rab10 levels in different brain regions (Fig. 2h).

## Discussion

The pathology of patients with LRRK2 variants is diverse, and the mechanism by which LRRK2 variants lead to neurodegeneration remains unclear. The current study reports a patient with PD harboring LRRK2 G2385R, a risk variant in the Asian populations, who exhibited a typical diffuse Lewy body pathology with NFTs and amyloid plaques, showing mild signs of neuroinflammation. NFTs and threads contain both 3-repeat and 4-repeat tau, which correspond to an “intermediate” level of AD pathological changes.

Several studies have indicated that LRRK2 is induced by IFN-γ and is involved in neuroinflammation^34–36^. LRRK2 is highly expressed in human peripheral blood neutrophils and microglia^37^ and is considered a modulator of neuroinflammation^38^. Microglia are also activated through a mechanism in which polyglutamine-binding protein 1 senses extrinsic tau by direct interaction and triggers an innate immune response by activating the cyclic GMP-AMP synthase-stimulator of the interferon gene pathway^39^. Despite this, the current case had mild gliosis as well as cases with LRRK2 R1441H^24^. This observation does not rule out the involvement of LRRK2 in neuroinflammation. Given the very slow disease progression and long disease duration in this case, it is possible that there was sufficient time for neuroinflammation to converge after neuronal loss. Studies have suggested that G2385R inhibits LRRK2 dimerization and destabilizes LRRK2 protein, while it enhances kinase activity^21,40^. This study confirmed prominent phosphorylation of Rab10 in LRRK2 G2385R patients, strongly suggesting that the G2385R variant enhances LRRK2 kinase activity in the human brain.

Phospho-Rab10 can reportedly be used as a surrogate marker for LRRK2 kinase activity; however, the pathological significance of the increase in phospho-Rab10 requires further research^41,42^. The lack of increase in phospho-Rab10 in the frontal cortex is striking, and at least two possible explanations exist. One is the possibility of higher phosphatase activity toward phospho-Rab10 in the frontal cortex. PPM1H, a protein phosphatase, is reportedly involved in the dephosphorylation of phospho-Rab10^43^. However, PPMIH expression is not particularly high in the frontal cortex, there may be an unidentified phosphatase(s)^44^. Another possibility is the presence of LRRK2 regulators. The expression of LRRK2 inhibitory molecules and activators may differ between the frontal cortex and other regions.

A recent structural analysis revealed that the LRRK2 kinase domain is surrounded by the ankyrin repeat, LRR, ROC, COR, and WD40 domains, and the LRR domain spatially surrounds and shields the ATP-binding cleft^4^. Another study reported that a nanobody, which recognizes a region within the WD40 domain close to G2385R, functions as a kinase activator and inhibits MLi-2-induced LRRK2 filament formation, probably by disrupting the interaction between the WD40 and the LRR hinge helix^45^. These structural inferences are consistent with our biochemical findings.

Although various studies have shown that LRRK2 may be involved in αS pathology, there still appears to be a gap between LRRK2 pathology and these hypotheses. For example, a study reported that LRRK2 G2019S exacerbated αS pathology in an αS transmission mice model and human pluripotent stem cell-derived neurons^46^, suggesting that LRRK2 variants may promote αS aggregation. Another study showed that LRRK2 promotes αS propagation through Rab35 phosphorylation^47^. We biochemically evaluated the phospho-Ser129 αS accumulation in various brain regions of the LRRK2 G2385R patient, and the results were consistent with our pathological observation. However, there was no correlation between the levels of phospho-αS and phospho-Rab10. Intriguingly, phospho-Rab10 was colocalized in AT8-positive tau, but not in amyloid plaques, in tauopathy, including AD^48^. Moreover, the accumulation of tau, but not amyloid plaques, positively correlated with αS pathology in patients with LRRK2 mutations^27^. LRRK2 purportedly regulates microtubule dynamics^15^, and may indirectly affect the turnover and propagation of tau^49,50^. These observations may suggest that phosphorylation of Rab10 by LRRK2 affects tau dynamics, followed by αS accumulation. Collectively, our findings and those of previous pathological studies of PD with LRRK2 variants support that LRRK2 is not directly involved in αS aggregation and accumulation, but rather, LRRK2 exacerbates the brain environment and promotes aging. Indeed, tau pathology and senile plaque characteristic of the aging brain were often observed in LRRK2 pathology^26,27^.

The limitation of this study is the inadequate clinical information available from the family members owing to their lack of cooperation. We could not determine whether the younger sister is a G2385R carrier. Thus, whether G2385R variant in this patient with PD directly caused neuronal cell death, or the Lewy body pathology cannot be concluded from this study alone. Studies have reported results contradictory to those of the present study on the kinase activity of LRRK2 G2385R, showing no increase or a decrease in the kinase activity, although they did not evaluate kinase activity with Rab10 ^22,23^. Therefore, the effect of the G2385R variant on kinase activity needs further verification. Our biochemical analysis using post-mortem brain samples failed to detect an enhanced kinase activity in LRRK2 R1441H and I2020T variants, as opposed to previous studies reporting that these mutations increase kinase activity in LRRK2^23,25,51,52^. A recent study to quantify phospho-Rab10 levels in brain autopsy samples reported the lack of correlation between phospho-Rab10 levels and LRRK2 genotypes^53^. This suggests that Rab10 phosphorylation in post-mortem brain may be variable depending on the condition of sample preservation or undetermined phosphatase activity^43,48^. Therefore, our results must be evaluated in light of this issue. Further accumulation of pathology data from patients with G2385R and other LRRK2 mutants would be important to elucidate the pathomechanism of LRRK2.

In conclusion, this is a rare autopsy report of a patient with PD harboring LRRK2 G2385R variant, showing elevated phosphorylation of Rab10, a substrate of LRRK2, and characteristic pathology as synucleinopathy. Our data provide evidence to support that chronic elevation of LRRK2 kinase activity may be factorial in accelerated brain aging rather than directly being involved in αS accumulation. These findings can be helpful for further understanding of the pathogenesis of *LRRK2*-linked PD.

## Methods

### Background information of patients and controls

The G2385R patient’s family was from the eastern area of Japan with no consanguinity reported. No other members in the G2385R family developed PD. We prepared samples from three patients with no PD as controls, four patients with sporadic PD, and two patients harboring LRRK2 R1441H or I2020T^29,30,54^. This study (M08-0477) was approved by the ethics committee of Juntendo University, Tokyo, Japan. Written consent was obtained from all the patients who were included in Supplementary Table 1.

### Clinical and genetic analysis

PD diagnosis was made using the Movement Disorder Society clinical diagnostic criteria for PD^55^. Brain MRI, melanin imaging MRI, SPECT with intravenous injection of N-isopropyl-p-[^123^I] iodoamphetamine, DAT-SPECT, and myocardial scintigraphy with [^123^I] MIBG were performed as neuroimaging examinations. Genomic DNA was extracted from peripheral blood samples using a standard protocol. We screened genes related to familial PD or dementia using target sequencing by Ion Torrent system (Thermo Fisher Scientific, Waltham, MA, USA); the panel (IAD103177_182) was set up to screen *SNCA*, *PARK2*, *UCHL1*, *PINK1*, *DJ-1*, *LRRK2*, *ATP13A2*, *GIGYF2*, *HTRA2*, *PLA2G6*, *FBXO7*, *VPS35*, *EIF4G1*, *DNAJC6*, *SYNJ1*, *DNAJC13*, *CHCHD2*, *GCH1*, *NR3A2*, *VPS13C*, *RAB7L1*, *BST1*, *c19orf12*, *RAB39B*, *MAPT*, *PSEN1*, *GRN*, *APP*, and *APOE*^56^. The identified variants were confirmed by Sanger sequencing. The panel for sequencing was designed with Ion AmpliSeq Designer (Thermo Fisher Scientific, https://www.ampliseq.com). We also collected genomic DNA from the brain tissues of 10 individuals, which included control cases, using QIAamp DNA Blood Midi Kit (QIAGEN, Hilden, Germany). LRRK2 exon 48 was sequenced using the Sanger method, as reported previously^2^. These genetic markers were genotyped by Sanger method as reported previously^57^.

### Neuropathological analysis

We obtained brain autopsies from the patients and carried out neuropathological examinations, comparing them to those of control cases. Brains were fixed with 15% neutral buffered formalin, and the selected tissues were embedded in paraffin. The paraffin embedded blocks were sliced at 6 μm thickness. Brain sections were stained with hematoxylin and eosin, Klüber-Barrera, methenamine-silver stain, Gallyas–Braak stain, and immunohistochemical staining for proteins related to neurodegenerative diseases. For immunohistochemistry, brain sections underwent antigen retrieval either by heat activation in a microwave oven or by reaction in formic acid, before being incubated with primary antibodies overnight at 4 °C. The primary antibodies used were as follows: anti-phospho-Ser129 αS (pSyn#64, monoclonal, 1:1,000 dilution, Wako, Osaka, Japan or EP1536Y, 1:400, Abcam, Cambridge, UK), anti-phospho-tau (AT8, monoclonal, 1:200, Thermo Fisher Scientific), anti-tau RD3 (8E6/C11, 1:100, Merck, Darmstadt, Germany), anti-tau RD4 (1E1/A6, 1:100, Merck)^58^, anti-4R tau (TIP-4RT-P01, 1:3,000, Cosmo Bio, Tokyo, Japan), anti-amyloid ß (1–42, polyclonal, 1:100, IBL, Gunma, Japan), anti-TH (TH-16, 1:2,000, Sigma-Aldrich, Merck), anti-phospho-TDP-43 (11–9, Ser 409/410, monoclonal, 1:3,000, Cosmo Bio), anti-GFAP (G-25-8-3, monoclonal, 1:500, IBL), and anti-ionized calcium-binding adapter molecule 1 (Iba1; polyclonal, 1:500, Wako). Immunosignals were visualized using the peroxidase-polymer-based method with a Histofine Simple Stain MAX-PO kit (Nichirei, Tokyo, Japan) and diaminobenzidine as the chromogen.

### Biochemical analysis using brain autopsies

Biochemical fractionation using human brain tissues was performed as described previously^59^. Briefly, 20 mg of the putamen dissected from each frozen tissue sample was homogenized in 200 μL Buffer A (10 mM Tris–HCl, pH 7.4, 0.8 M NaCl, 1 mM EGTA, 10% sucrose) with a complete protease inhibitor cocktail (Merck) and spun at 100,000 *g* for 20 min at 4 °C. The supernatant was retained as a Tris buffer-soluble fraction. The pellet rinsed with 200 μL Buffer A was homogenized in 150 μL Buffer A containing 1% Triton X-100 and incubated for 30 min at 37 °C. After centrifugation at 100,000 *g*, the supernatant was retained as a Triton X-soluble fraction. The pellet was then rinsed with 150 μL Buffer A and further homogenized in 100 μL Buffer A containing 1% sarkosyl, incubated at 37 °C for 30 min and spun at 100,000 *g* for 20 min. The supernatant was retained as a sarkosyl-soluble fraction. The sarkosyl-insoluble pellet was resuspended in Laemmli sodium dodecyl sulfate (SDS) sample buffer and used as a sarkosyl-insoluble fraction. Tris buffer-soluble, Triton X-soluble, sarkosyl-soluble, and sarkosyl-insoluble fractions were subjected to SDS-polyacrylamide gel electrophoresis/western blotting. These sequential extraction procedures were performed repeatedly by using 20 mg of the frontal, temporal, occipital, entorhinal cortexes, and cerebellum dissected from each frozen tissue sample. All blots derive from the same experiment and were processed in parallel. The full-length uncropped images of western blot results were shown in Supplementary Fig. 2.

## Supplementary information

Supplementary Information

## Data Availability

The data generated during and/or analyzed during this study are available from the corresponding authors on reasonable request.
