# Supplementary material for: Pathophysiological evaluation of the LRRK2 G2385R risk variant for Parkinson’s disease
Source: NPJ Parkinsons Dis. 2022 Aug 5;8:97. doi: 10.1038/s41531-022-00367-y (PMC9355991; doi:10.1038/s41531-022-00367-y)
Supplement: Supplementary file 1 — Supplementary Information [file 41531_2022_367_MOESM1_ESM.pdf]

Supplementary Information:

Pathophysiological evaluation of the *LRRK2* G2385R risk variant for Parkinson's disease

Toshiki Tezuka[1,2], Daisuke Taniguchi[1], Mariko Sano[1], Tomoyo Shimada[1], Yutaka Oji[1], Taiji Tsunemi[1], Aya Ikeda[1], Yuanzhe Li[1], Hiroyo Yoshino[3], Jun Ogata[4], Kahori Shiba-Fukushima[5], Manabu Funayama[1,3,6], Kenya Nishioka[1], Yuzuru Imai[1,4], Nobutaka Hattori[1,3,4,5,6,7]

[1]Department of Neurology, Juntendo University School of Medicine, Tokyo 113-8421, Japan

[2]Department of Neurology, Keio University School of Medicine, Tokyo 160-8582, Japan

[3]Research Institute for Diseases of Old Age, Juntendo University Graduate School of Medicine, Tokyo 113-8421, Japan

[4]Department of Research for Parkinson's Disease, Juntendo University Graduate School of Medicine, Tokyo 113-8421, Japan

[5]Department of drug development for Parkinson's disease, Juntendo University Graduate School of Medicine, Tokyo 113-8421, Japan

[6]Center for Genomic and Regenerative Medicine, Graduate School of Medicine, Juntendo University, Tokyo 113-8421, Japan

[7]Neurodegenerative Disorders Collaborative Laboratory, RIKEN Center for Brain Science, 2-1-Hirosawa, Wako-shi, Saitama 351-0198, Japan

Contents:

Supplementary Figures 1-2
Supplementary Table 1

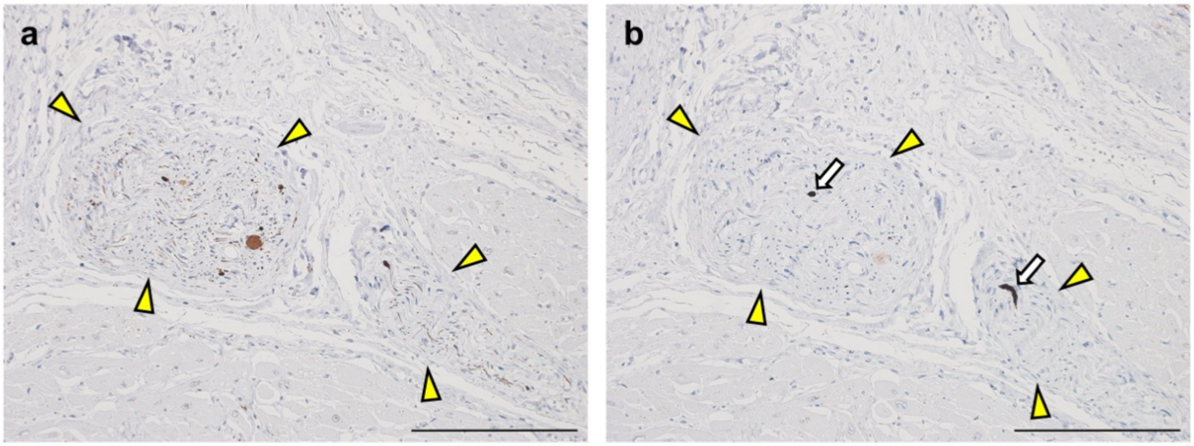

Supplementary Figure 1. Denervation of TH-positive sympathetic fibers (**a**) and accumulation of p-αS (**b**) in the heart. Serial sections were immunostained with anti-TH (**a**) and anti-p-αS (**b**). Arrowheads and arrows indicate sympathetic fibers with a marked reduction in anti-TH immunosignals and anti-p-αS-positive inclusions, respectively. Scale bars: 200 μm.

Figure 2a

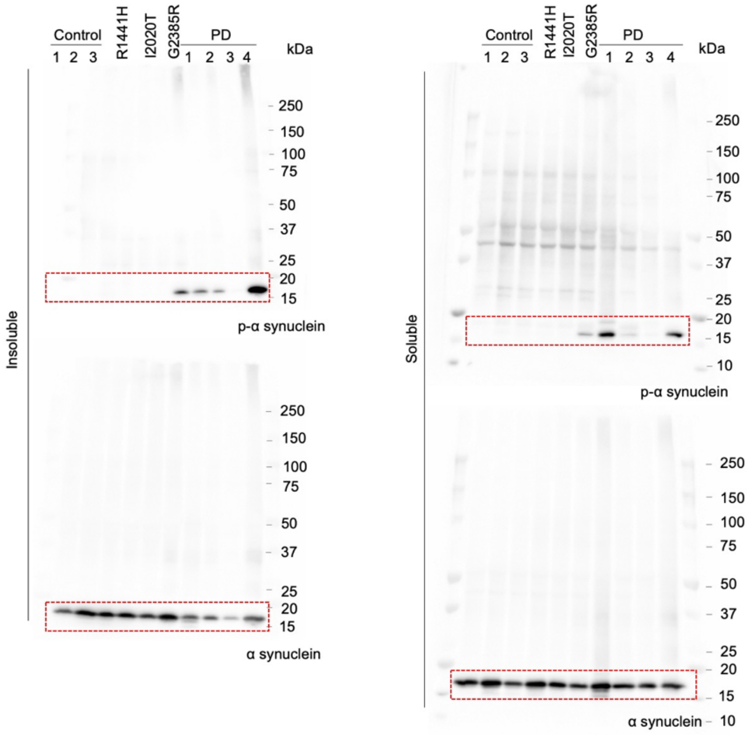

Figure 2g

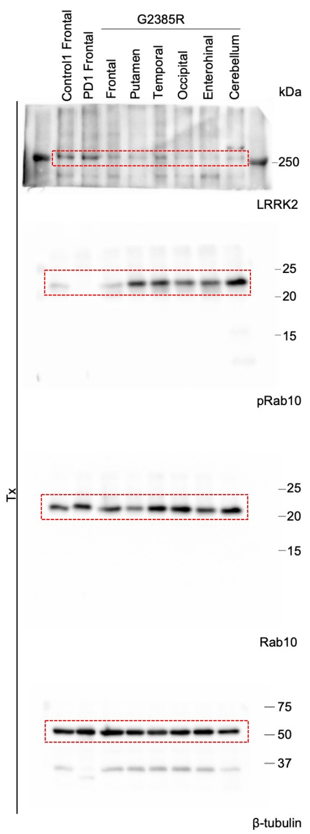

Figure 2c

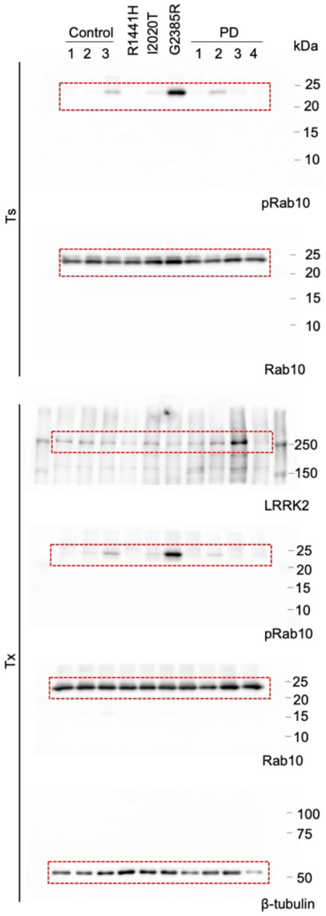

Figure 2e

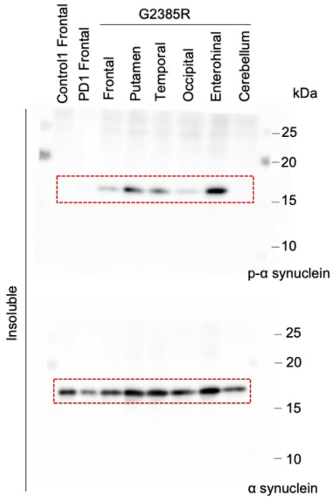

Supplementary Figure 2. The full-length uncropped images of western blot results from Figure 2.

Case	Age at death (years)	Sex	Disease duration (years)	Brain weight (gram)	PMI (min)	Lewy pathology type	Braak staging	AT8 staging	Thal phase
Control-1	78	M	-	1350	725	-	-	1	1
Control-2	82	F	-	1400	488	-	-	3	2
Control-3	68	M	-	1300	2440	-	-	2	1
PD-1	69	F	17	1370	615	Diffuse neocortical	5	2	2
PD-2	76	M	20	1200	914	Limbic	4	2	0
PD-3	81	M	12	1180	145	Limbic	4	2	0
PD-4	87	F	10	1490	2683	Diffuse neocortical	5	1	4
LRRK2 R1441H	74	M	14	1350	946	-	-	1	1
LRRK2 I2020T	84	F	32	1060	598	-	-	1	0
LRRK2 G2385R (This study)	72	F	32	1297	958	Diffuse neocortical	5	4	3

Supplementary Table 1. Clinical information of brain autopsies.
PMI, postmortem interval.
